# Comprehensive structural model for the evaluation of HME humidification properties

**DOI:** 10.1038/s41598-025-17916-z

**Published:** 2025-09-17

**Authors:** Ingolf Meineke, Klaus Züchner

**Affiliations:** 1 Department Clinical Pharmacology, University Medical Centre, Robert-Koch-Str. 40, D-37075 Göttingen, Germany; 2 Department Anaesthesiology, University Medical Centre, Robert-Koch-Str. 40, D-37075, Göttingen, Germany

**Keywords:** Heat and moisture exchanger, HME, LE-HME, Performance, Efficiency, Method validation, Model calculator program, Health care, Medical research, Techniques and instrumentation, Theory and computation

## Abstract

HMEs (heat and moisture exchangers) are employed in clinical practice to humidify the inspired air in intubated patients. We aimed to develop and experimentally validate a mass based model describing the water input-output equilibrium in the presence of HMEs. The model should serve as a universally applicable basis for the evaluation of the fundamental HME humidification properties performance (mg reversibly stored per breath) and efficiency. A plug flow model was designed using three different locations (gas inlet, moisture benefit and water output) for inspiratory-expiratory flow and humidity measurements. We developed a complete set of algorithms for the calculation of all relevant metrics. Measurements also assessed humidity dependent volume changes. We proved the concept through the validation of humidity predictions without HME in the test setup at target humidities of 37 and 44 mg/l respectively with the inspiratory volume of 0.25, 0.50 and 0.75 l. The concept was applied in the evaluation of a random sample of HMEs. Differences between prediction and measurements ranged between − 1.89 and 2.24 mg/l. For moisture benefit a mean difference of 0.15 mg/l (sd 0.69) was found and for water output of -0.48 mg/l (sd 0.98). Humidification caused a significant volume increase up to 6.6% with respect to the inspired dry air. HME performance results of five HME brands ranged from 5 to 30 mg overall. Performance results derived from moisture benefit and water output agreed within 5% of the common mean. Efficiency results can be above 80% over the whole measurement range or below 20% at high water load depending on the HME brand. Thus, HMEs can be sorted by registering efficiency at the upper bound of the tidal volume range. HMEs for laryngectomized patients (LE-HMEs) showed much lower performances between 2 and 5 mg per breath due to their small size. A novel comprehensive research environment, both theoretical and experimental, enables the assessment of HME properties and gives qualitative and quantitative definitions of HME performance and efficiency for clinical use.

**Supplementary Information:**

The online version contains supplementary material available at 10.1038/s41598-025-17916-z.

## Introduction

HMEs (heat and moisture exchangers) have been used in clinical practise for several decades.

During this time, much work has been devoted to the task of providing reliable information to the clinician regarding the contribution of an HME to the humidification of inspired air. The upper airway provides 75% of the heat and moisture supplied to the alveoli^1^. When bypassed the humidifier needs to supply the missing heat and moisture. Various measurement principles have been applied to the HME assessment task; among them direct weighing of the HME^2^, weighing of the test apparatus^3–6^, direct measurement of absolute and relative humidity^7–9^, psychrometry^10^ and measurement of temperature differences^11^.

Many HME tests were laboratory based bench tests^3,4,10,12^, while other authors endeavoured to adapt test conditions as close as possible to the clinical situation^9,13,14^.

As heterogeneous as the experimental approaches are the target quantities reported to describe the effect and contribution of HMEs to air humidification. We find e.g. moisture output, water loss, efficiency, efficacy, moisture return, delivery, HME performance and capacity. The metric used in a particular publication is not always unanimously defined.

This multitude of test procedures and metrics makes it difficult to compare figures across the results published in the relevant literature with confusing outcomes.

In addition to bare measurements of humidity related variables structural models have been described by several authors^3–6,8,10,15–18^ with the aim to elaborate and define essential experimental parameters in HME testing.

Again, all these efforts have not yet resulted in an internationally agreed upon and commonly used standardized procedure for HME testing nor in a unanimously defined criterion in clinical practise for the humidification an HME achieves. Both are required for informed decision making by the clinician.

Our aim in this work was to make a proposition for a unified nomenclature in HME testing. The fundamental HME property consists in the ability to store a fraction of the water vapour load the device is exposed to during expiration. *HME performance* then equals the reversibly stored amount of water [mg] under given conditions rather than a concentration. *HME efficiency* is the ratio of the mass of water reversibly stored in the HME and the net mass of water entering the HME per breath. Both values should be accessible to any laboratory regardless to the equipment in use.

We also present a mass based structural model with standardized implementation introducing moisture benefit (*mb*, mg/l) as a measurable quantity in addition to water output (*wo*, mg/l). Specific detailed information is included as an example to allow implementation in any laboratory if desired. Measurement results from five selected HME brands at two humidification levels with three tidal volume settings are presented.

The structural model is accompanied by a comprehensive mathematical framework that allows the calculation of all important quantities in the model (See appendix for details). The versatile applicability allows the evaluation of experimental data based on the assessment of more than one combination of measurable quantities i.e. humidity, volume, flow, temperature and pressure. For the first time volume changes in the gas stream due to water addition and removal are taken into consideration in this framework.

We show also the applicability of the model to the testing of HMEs for laryngectomized patients. These HMEs are different from conventional HMEs since they are only used for spontaneously breathing patients and have no machine port for connection with a ventilator. Measurement of *mb* allows direct access to HME performance information even in these HMEs. Data from eight LE-HMEs are included.

## Methods

### Model structure

**Fig. 1:**
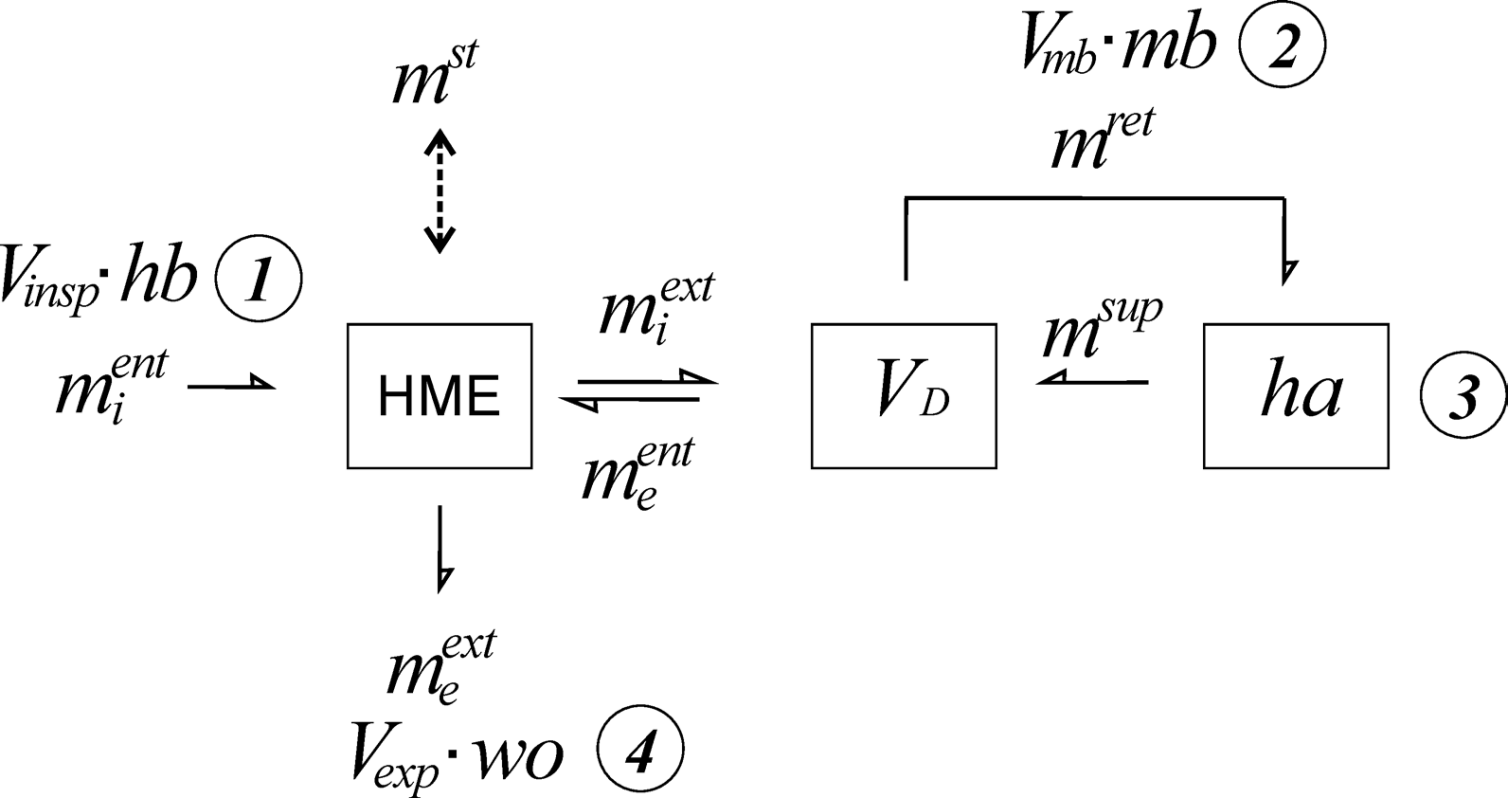
Structural model for assessment of HME performance. $\:{{m}_{i}^{ent}}_{}^{}$ mass entering the HME during inspiration (position 1, measurement of *hb* and $\:{V}_{insp}$), $\:{m}^{st}$ mass reversibly stored in HME, *HME performance*, $\:{m}_{i}^{ext}$ mass leaving the HME during inspiration and entering the dead space (V_D_), $\:{m}_{e}^{ent}$ mass leaving the dead space and entering the HME during expiration (water load), $\:{m}^{ret}$ mass leaving the dead space during inspiration and entering the humidifier, (position 2, measurement of *mb* and *V*_*mb*_), $\:{m}^{sup}$ mass leaving the humidifier during expiration (position 3, pre-set target humidity), $\:{m}_{e}^{ext}$ mass leaving the HME during expiration (position 4, measurement of *wo* and *V*_*exp*_). $\:{m}^{add}={m}_{e}^{ext}-{m}_{i}^{ent}$ mass added in the humidifier, $\:eta=\eta\:=\frac{{m}^{st}}{{{m}_{e}^{ent}-m}_{i}^{ent\:\:}}$ *HME efficiency.* For additional information see text.

The model structure (Fig. 1) was based on the model described by A. Wilkes in his thesis^3^. As an essential extension a separate arm denoting the amount returned (*m*^*ret*^) was added. In order to make use of the law of mass conservation in the mathematical treatment the quantities of water moving from one location in the model to another are expressed as masses in mmol if not otherwise pointed out. Moreover, the fact was included that the addition of water vapour to the gas stream during inspiration and the removal of water vapour during expiration result in volume changes. Measurements were planned at four location in the model. The volume of air inspired (*V*_*insp*_) and its humidity (*hb*) were assessed at position 1. At position 2 the volume leaving the HME during inspiration (*V*_*mb*_) and the corresponding humidity (*mb*) were measured. The target humidity (*ha*) at position 3 was set in the humidifier (see model implementation). Finally, the volume leaving the HME during expiration (*V*_*exp*_) and the corresponding humidity (*wo*) were taken at position 4. The geometric volume of the dead space (*V*_*D*_) was determined without HME in place. As a rule of thumb this quantity should be less than 20% of the inspired volume.

### Model properties

The dead space (*V*_*D*_) is the only intrinsic parameter to be ascertained, i.e. it depends on the model implementation. The remaining parameters required for a comprehensive determination of all mass quantities in the model are the target humidity (*h*a) which must be set, the humidity in the inspired gas (*hb*) and an arbitrary combination of one concentration measurement (*mb* or *wo*) and one volume measurement (*V*_*insp*_, *V*_*mb*_ or *V*_*exp*_). With such a set of data, the model is fully determined. Additional measurements lead to an overdetermined set of variables, but can be used to check for model consistency.

Under the assumption of plug flow conditions during the breathing cycle in steady state all masses defined in the model can be readily calculated from a set of measurements and the mandatory parameters as stated above to evaluate HME performance. In addition for a test rig without HME, expectations can be predicted for water output and moisture benefit measurements to asses suitability.

If for instance *V*_*mb*_ and *mb* are available as measurements the quantity $\:{m}^{st}$ is obtained as follows (masses and volumes are expressed as molar quantities and concentrations as molar fractions^5^):

$$\:{m}^{st}=\left\{\left[\frac{mb*{V}_{mb}-ha*{V}_{D}}{1-\frac{{V}_{D}}{{V}_{mb}}}\right]-hb*{V}_{mb}\right\}/(1-hb)$$

(See appendix: Evaluations).

With the knowledge of $\:{m}^{st}$ the remaining unknowns in the model (Fig. 1) can directly be calculated. The complete set of equations (see appendix) has been implemented in a computer program called ‘HME-calculator’ which has been used for all calculations in this work. The program is available from the authors upon request.

**Fig. 2:**
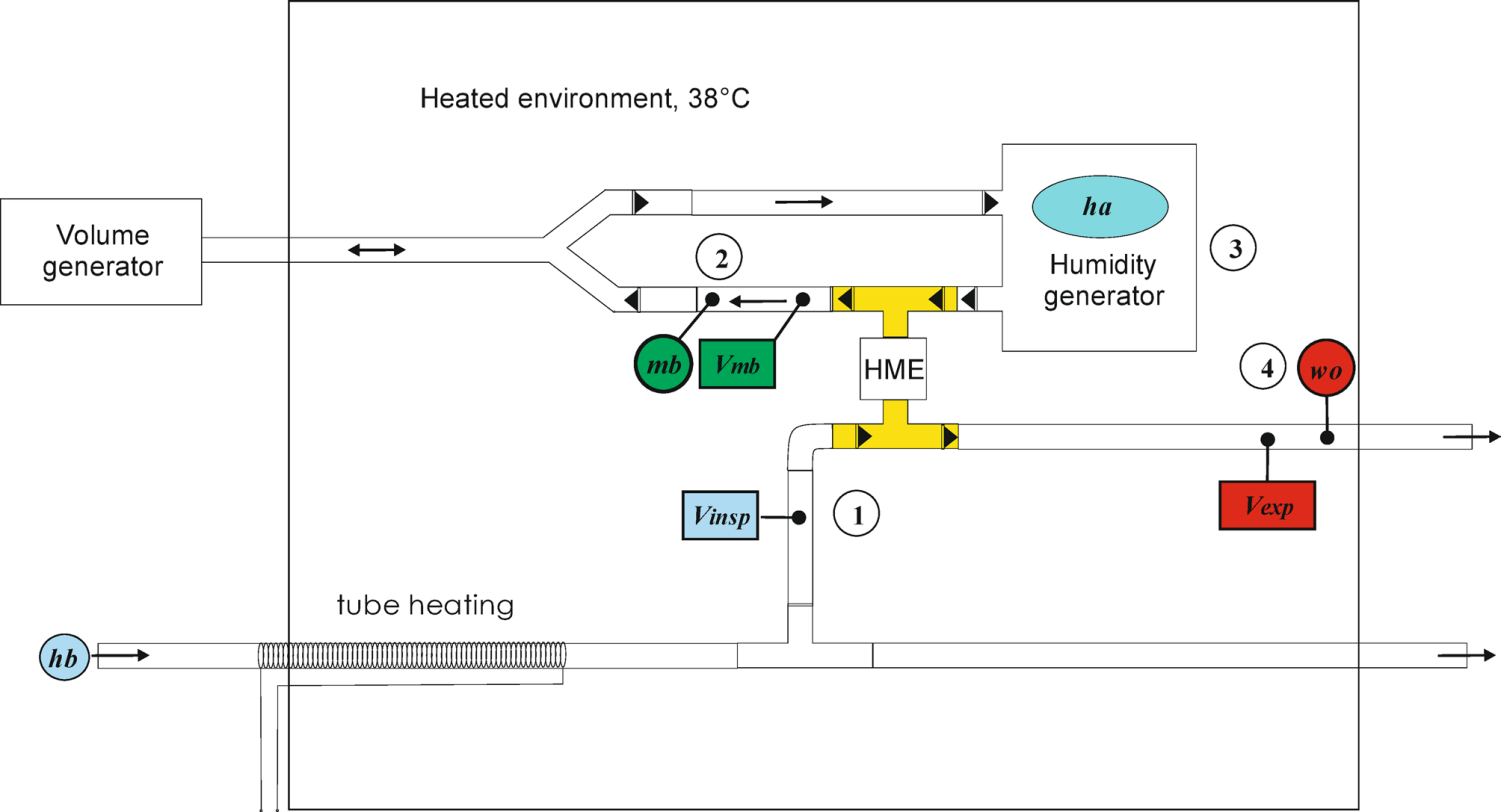
Test rig construction scheme describing flow pathways (arrows) and measuring positions. *V*_*insp*_, *hb*: position #1, *V*_*mb*_, *mb*: position #2, *ha*: position #3, *V*_*exp*_, *wo*: position #4. Triangles indicate one way valves.

### Model implementation

From the scheme in Fig. 1 the actual implementation was built as depicted in Fig. 2. The model used as a body a temperature controlled cabinet (Air Shield Vickers Infant Incubator, Heinen & Löwenstein, Bad Ems, Germany). The flow through the model (15 breath per minute, sinusoidal flow I: E 1:1, range 0.2− 0.8 l per breath, similar to the recommendations in^4^ was controlled by a volume generator type LS 1500 (Draeger, Lübeck, Germany). The generated inspiratory volume (*V*_*insp*_) was measured in the inspired dry air with a calibrated volume meter (Ventilator tester EKU ViP, EKU Elektronik GmbH, Leiningen, Germany). An adjustable external air flow up to 60 l/min, humidity (*hb*) less than 0.1 mg/l in excess to the maximum inspiratory flow was provided by the central gas supply of the university hospital if not specified otherwise. The HME under investigation was placed between two T-pieces with directional valves (Intersurgical, Sankt Augustin, Germany, part no.1954000) to separate inspiration and expiration flows. Flow and humidity were measured and recorded in the moisture benefit limb with a pneumotachograph(PTG) according to Fleisch^19^ and a fast responding sensor (FT 202 M ZSK Systemtechnik, Katlenburg, Germany)^7^.

Humidification of the expired air to typical pre-set saturation temperatures (34 °C and 37 °C) found in the literature was achieved with a humidity generator HumiCare Delta (Gründler Medical, Freudenstadt, Germany). The humidifier is equipped with a length of actively heated tubing. This tubing was placed directly at the air inlet into the cabinet housing.

During expiration the air flow was directed into the humidifier and passed then into the exhaust tubing through the T-pieces, and the HME location. Flow and humidity in the expired air were measured and recorded with a second set of sensors as in the moisture benefit limb.

The humidity sensors measure water partial pressure and operate independently on the gas used. They were calibrated against a thermo-hygrometer (testo 625, Testo, Lenzkirch, Germany). The PTGs were calibrated against the EKU volumeter.

Data acquisition was via suitable analogue to digital converters at 10 Hz. Data were stored as csv type files. Water output and moisture benefit were calculated breath for breath from flow and humidity records. The corresponding volumes *V*_*mb*_ and *V*_*exp*_ were also calculated from the flow measurements.

The test system reached equilibrium after commissioning within less than an hour. After an HME change, a new equilibrium was set in less than ten minutes.

## Results

### Test rig suitability

**Fig. 3:**
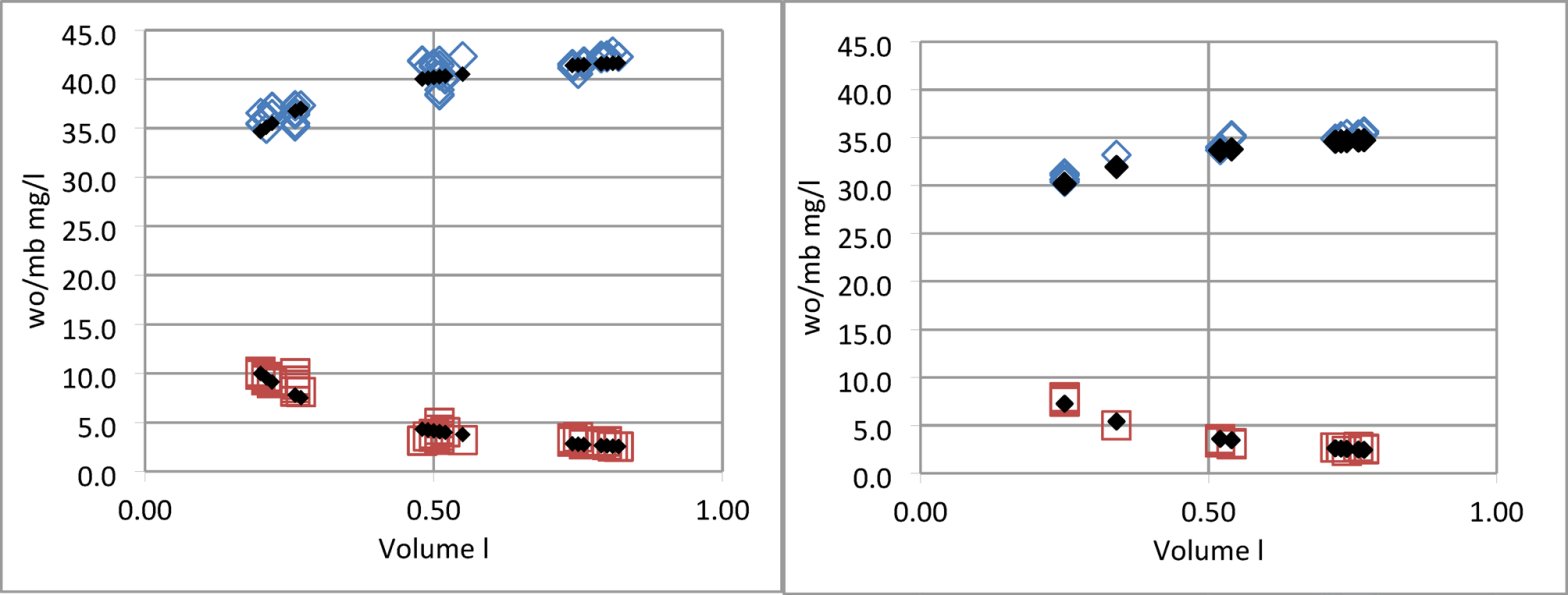
Water output and moisture benefit without HME as a function of volume inspired. Left panel: *ha* = 44 mg/l, right panel: *ha* = 37 mg/l. Blue diamonds water output measurements mg/l, red squares moisture benefit measurements mg/l. Black symbols corresponding predictions.

The suitability of the test rig was assessed by measuring *wo* and *mb* without HME (Fig. 3). The measurement results were compared with the corresponding predictions on the base of the test rig model in Fig. 1. Predictions were obtained with settings *V*_*D*_ = 30 ml, ambient temperature 38 °C, carrier gas humidity *hb* = 0, and the appropriate values for humidifier target humidity and the inspired volume. Detailed arithmetic prescriptions are outlined in the appendix.

Precision of humidifier temperature was +/- 0.1 °C, the corresponding humidity ranged between 37.0 and 37.5 mg/l at 34 °C and between 43.7 and 44.2 mg/l at 37 °C. This imprecision results in a variability of +/- 0.5 mg/l in the predictions of moisture benefit and water output. The overall differences between measured and predicted values ranged from − 1.89 to 2.24 mg/l with mean − 0.15 and standard deviation 0.69 mg/l for moisture benefit measurements while water output results had a mean of -0.48 and a standard deviation of 0.98 mg/l.

Thus, measured and predicted values were in satisfactory agreement over the whole ranges of humidity and tidal volume, indicating the applicability of the structural model.

### Volume effects

**Fig. 4:**
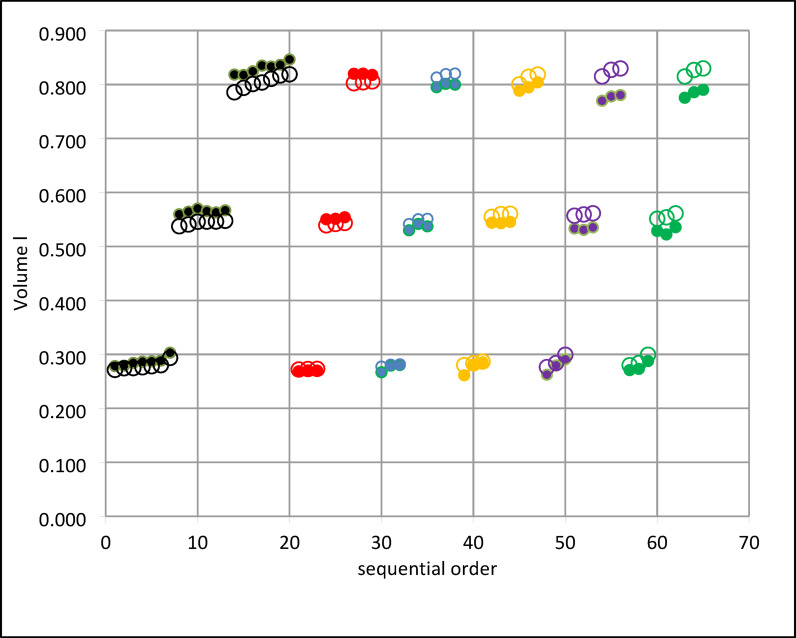
Volume *V*_*mb*_ and *V*_*exp*_: systematic differences at target humidity 44 mg/l. Open circles *V*_*mb*_, full circles *V*_*exp*_. Colors refer to different HME brands: black: base (no HME), red: HME 1, blue: HME 2, khaki: HME3, purple: HME 4, green HME 5. For each HME brand *V*_*mb*_ and *V*_*exp*_ are contrasted to emphasize the differences in water content. For each HME from left to right (sequential order) *V*_*mb*_ at target volume 250 (3 points), *V*_*mb*_ at target volume 500 (3 points) and *V*_*mb*_ target volume 750 (3 points). Base results comprise all available observations.

The addition of water vapour to the dry carrier gas results in an expected volume increase of 6.6% of the inspired volume at the exit of the humidifier, when the gas is water vapour saturated to 44 mg/l. A part of the addition occurs in the HME if present. The final distribution of the additional volume between *V*_*mb*_ and *V*_*exp*_ depends on the performance of the HME in the system. Our measurements in Fig. 4 show clearly that without HME (base) a maximum increase in *V*_*exp*_ is registered and that *V*_*exp*_ is always larger than *V*_*mb*_. With increasing HME performance (see below) *V*_*mb*_ is increased with decreasing *V*_*exp*_ and the order is even reversed for particular HMEs. Volume results should therefore always state the measurement location (see Fig. 1).

### Moisture benefit and water output measurements

**Fig. 5:**
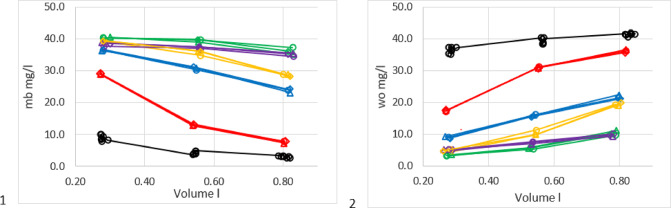
Moisture benefit and water output as a function of the corresponding volumes at 44 mg/l humidification level. Black: base (no HME), red: HME 1, blue: HME 2, khaki: HME3, purple: HME 4, green HME 5. For each HME type three specimen were included. Base results comprise all available observations: (**a**) Moisture benefit, target humidity 44 mg/l, (**b**) Water output, target humidity 44 mg/l.

Both, humidification level and tidal volume determine the amount of water reversibly stored in the HME. This is reflected in the moisture benefit and water output measurements (Fig. 5). The observed concentration decreases for moisture benefit with increasing tidal volume at given humidity levels. This decrease is differently large for each HME in the test series. The contribution of the dead space is depicted in the base line results. The decrease in moisture benefit caused by an increase in tidal volume is counteracted by an increase in humidity level.

Water output results data are complimentary to moisture benefit observations in as far as concentrations increase with increasing tidal volume. Apart from this distinction, both metrics contain the same information and on subsequent evaluation lead to identical conclusions.

All HME measurements reflect intraday variability, base measurements comprise both, intra-day and inter-day imprecision.

### HME performance evaluation and HME efficiency

**Fig. 6:**
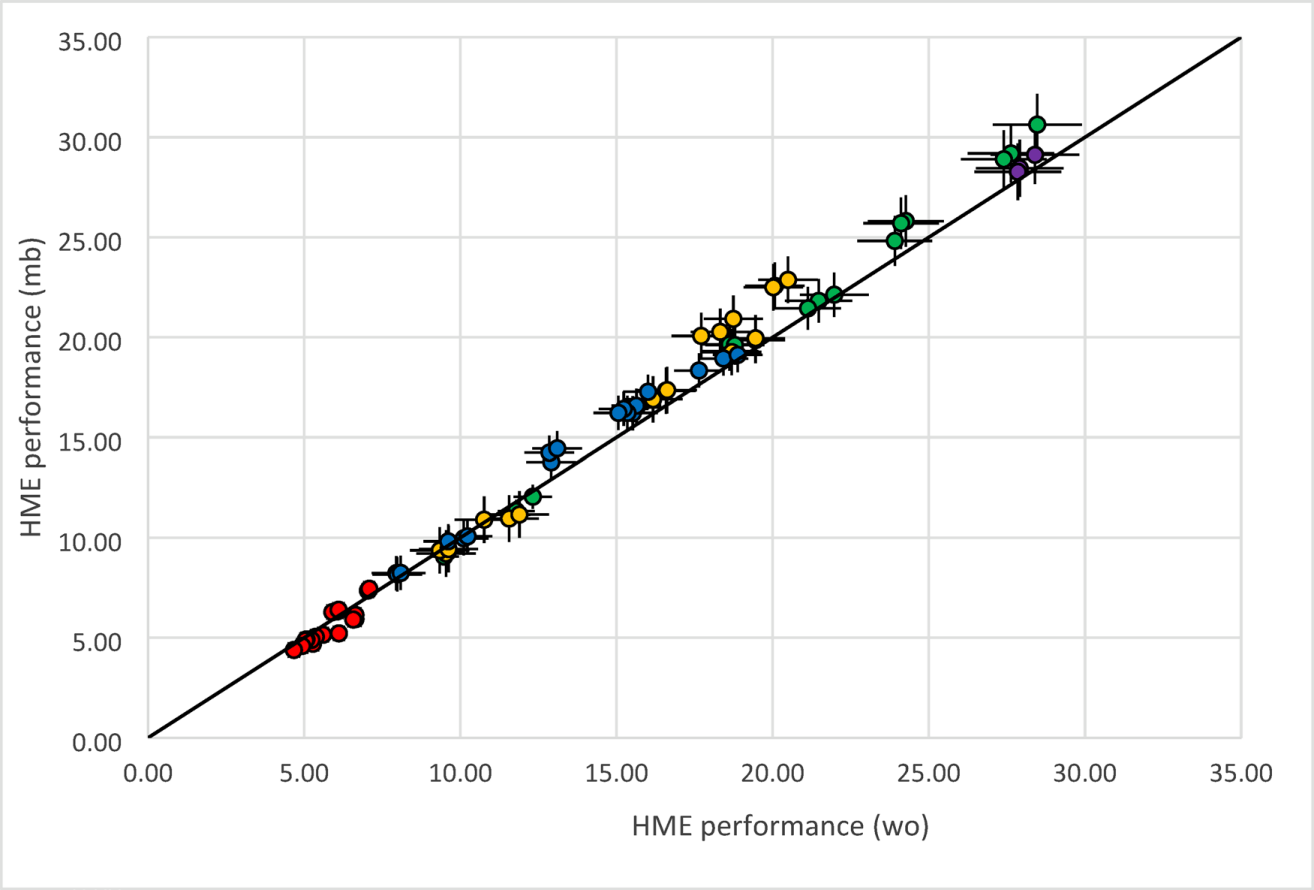
HME Performance [mg] calculated from *mb* and *wo* and the related volumes at both target humidities, (44 and 37 mg/l) for 3 specimen of 5 HME types. Horizontal and vertical bars indicate 5% range of the corresponding data point.

HME performance, defined as mass of water reversibly stored ($\:{m}^{st}$), and the mass entering during expiration ($\:{m}_{e}^{ent}$) were calculated (see appendix Fig. 11a-c) from the directly measured volume, moisture benefit and water output data. In Fig. 6 HME performance estimates obtained from moisture benefit and water output data in the same experiment are plotted against each other. The figure comprises all observations in all HMEs and all tidal volumes at both target humidities. The mean variability of performance results was 4.1% based on water output measurements and 1.5% with the moisture benefit data. In Fig. 6 an estimated propagated variability of 5% for the HME performance metric was used to illustrate the goodness of the fit to the expected identity line.

**Fig. 7:**
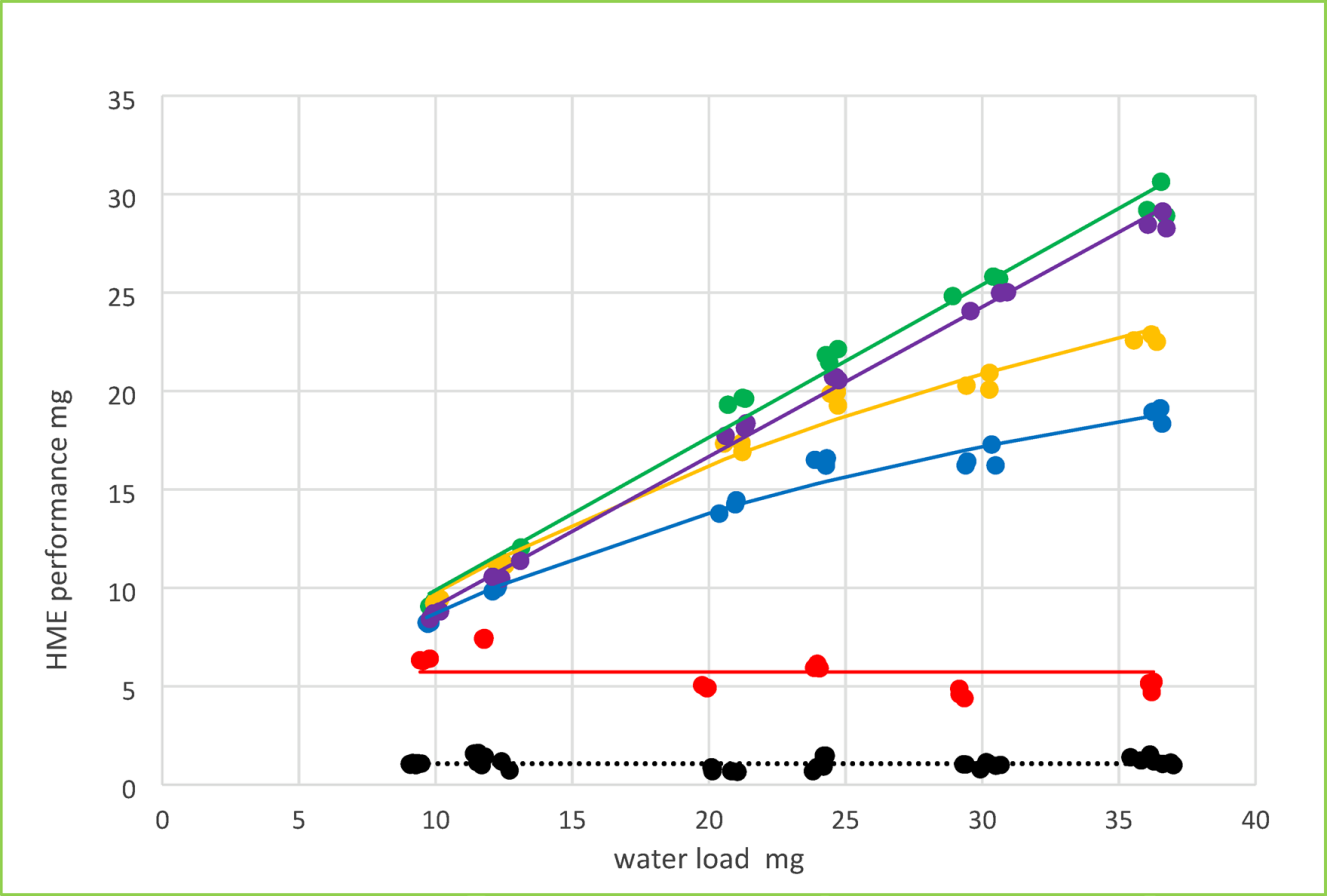
HME performance as a function of the amount entering the HME based on moisture benefit data. Black: base (no HME), red: HME 1, blue: HME 2, khaki: HME3, purple: HME 4, green HME 5. For each HME type three specimen were included. Base results comprise all available observations. HME performance and water load ($\:{m}_{e}^{ent}$) values were calculated as detailed in the appendix.

HME performance depends on tidal volume and target humidity. By plotting HME performance against mass entered during expiration (water load, $\:{m}_{e}^{ent}$), the separate influences of humidity level and volume were unified and all observations could be placed in one diagram (Fig. 7).

We discern three HME performance classes: linear, asymptotic and constant over the interval assessed. In the linear case increases the HME performance proportional to the mass entering. In the second case, a capacity limit (maximum amount storable) becomes visible. The HME performance increases less and less with increasing mass entering. This capacity limit has apparently reached in the third case. Therefore, the observed HME performance becomes independent on the mass entering the HME.

**Fig. 8:**
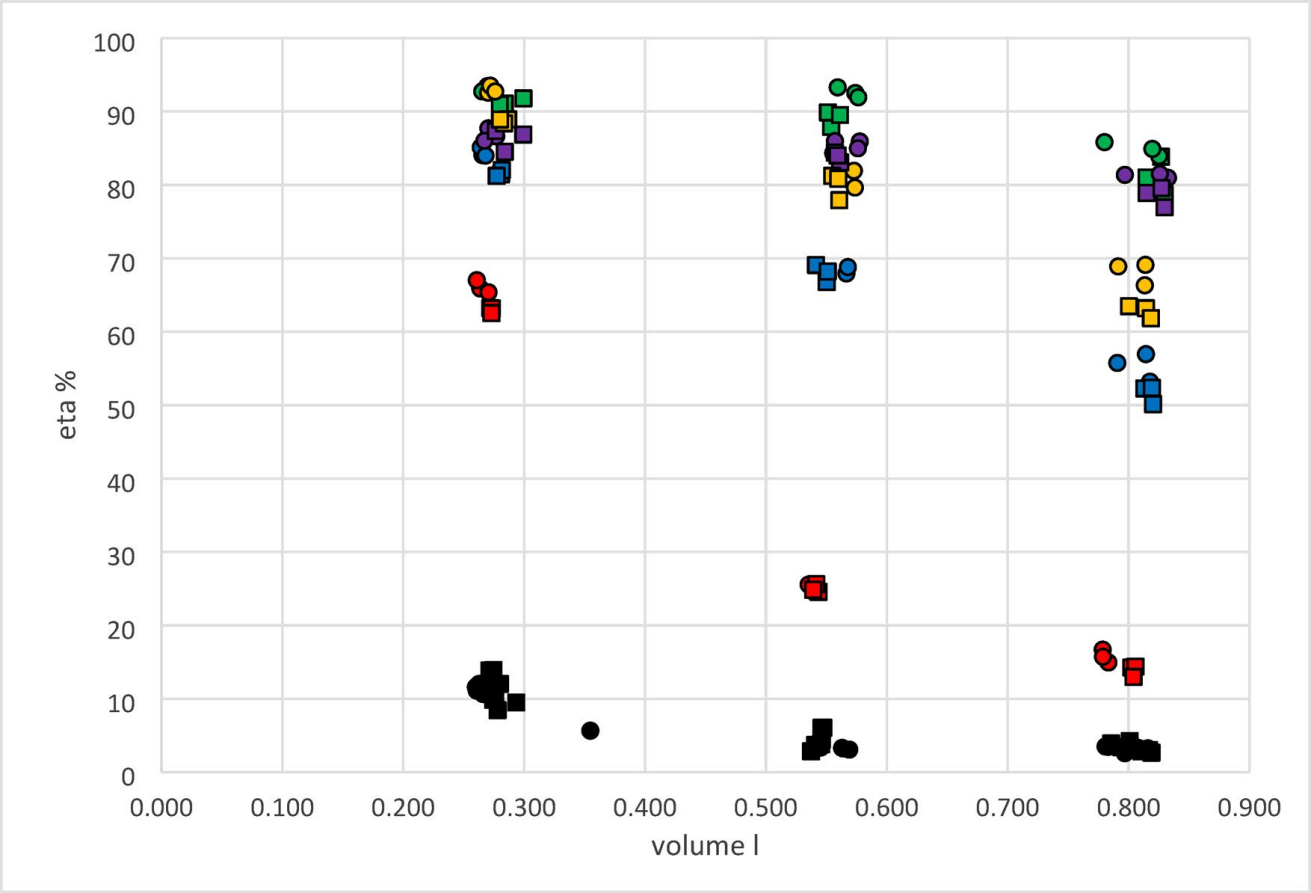
HME efficiency as a function of volume (*V*_*mb*_). Black: base (no HME), red: HME 1, blue: HME 2, khaki: HME3, purple: HME 4, green HME 5. For each HME type three specimen were included. Base results comprise all available observations: squares, target humidity 44 mg/l, circles, target humidity 37 mg/l.

HME efficiency was calculated (see appendix Fig. 11a-c) from HME performance and water load entering the HME under the conditions indicated. The figure shows a marked dependence of the efficiency on the tidal volume while the influence of the target humidity appears to be limited. The efficiency is nevertheless generally higher with the lower target humidity. On the basis of the information displayed in Fig. 8 HME assessments can be made by using the upper volume limit together with the corresponding characteristic efficiency. At lower volumes this efficiency is always maintained or even exceeded. For instance, HME 2 (khaki) possesses a guaranteed efficiency greater 60% with tidal volumes up to 0.8 l.

### Performance of LE-HMEs

**Fig. 9:**
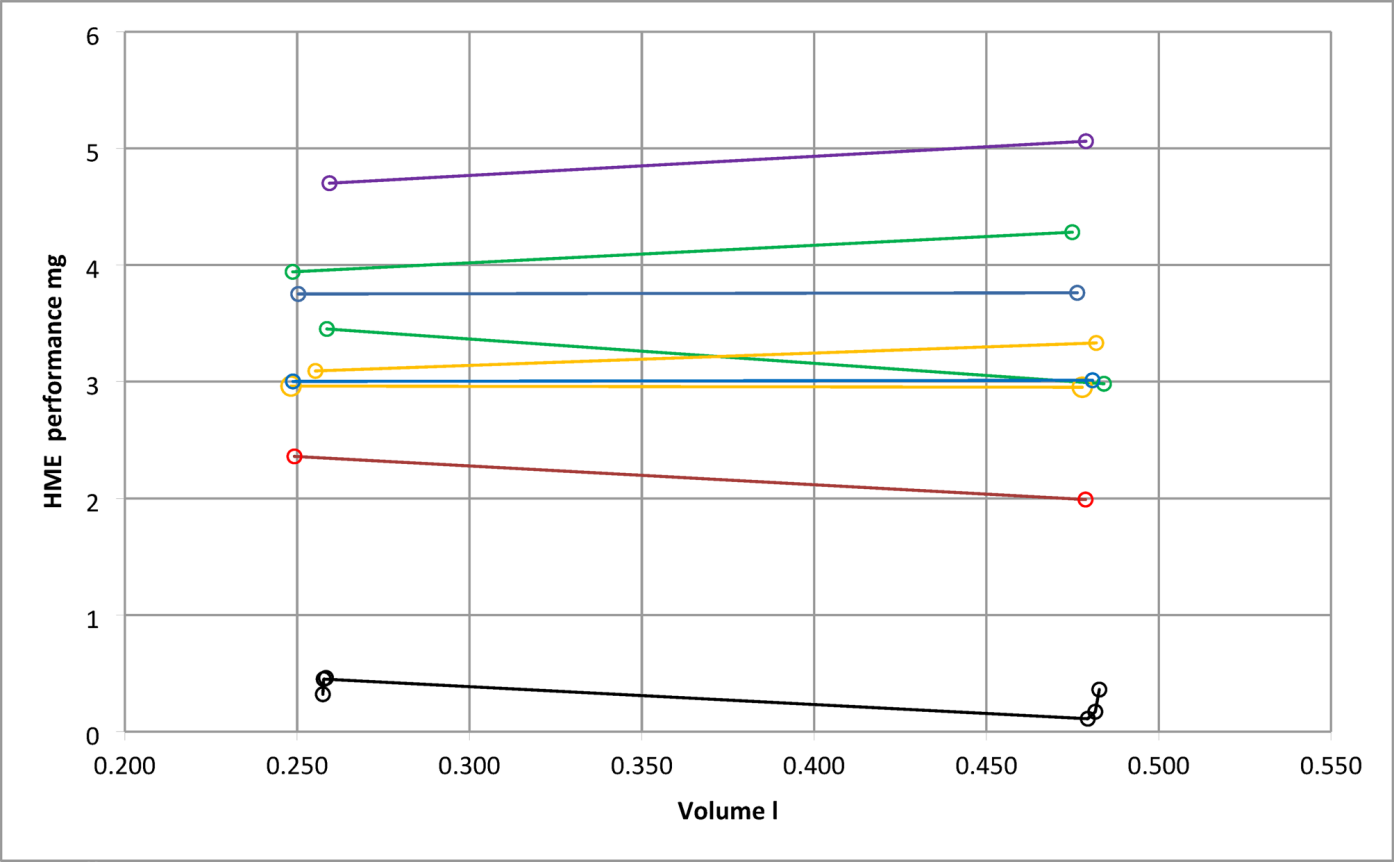
HME performance of LE HMEs as a function of volume (*V*_*mb*_). Black: base (no HME), red: HME 1, blue: HME 2, khaki: HME3,7, purple: HME 4, green HME 5,8.

In contrast to conventional HMEs which are mostly employed in operation theatres and ICUs LE-HMEs are intended for use in perambulating patients in common day situations. These HMEs must be considerably smaller than their counterparts and will typically inspire ambient air. Evaluation of performance was achieved with the moisture benefit and *V*_*mb*_ data (Fig. 9) since water output measurements were not available with LE-HMEs since LE-HMEs have no machine port.

LE-HMEs have a performance of between 2 and 5 mg per breath. Experimental conditions in case of LE-HME measurements were different in as far as carrier gas containing 10 mg water per litre was used. These results show that the test configuration used in this work can universally be employed to assess HME performance.

## Discussion

In contrast to a heated humidifier, a HME can only return humidity it previously has received, but cannot supply additional water. The results presented in this work provide a solid basis for future HME evaluations with regard to humidification properties. We have detailed a novel mass based model for the assessment of HME performance. On the basis of the model, we have suggested a proposition for the unequivocal definition of the terms performance and efficiency. A clinician can make an informed decision on which HME to use in a specific situation in intensive care and anaesthesia while an engineer can reliably generate this information.

### Suitability of test set-up

The prediction mode of the model was used (see appendix, Fig. 11b ) to assess the suitability of the test rig. Expectations for moisture benefit and water output with no HME were calculated with the inspired volume as input. The results (Fig. 3) show clearly that water output and moisture benefit measurements can be accurately be forecasted with a good variability. We have shown the complete set of measurements and consequently refrained from adding error bars. The suitability of the test rig was thus confirmed using only physical-chemical laws and mass conservation principles.

Prior investigations^3,9^ have used similar setups, they have however not taken the next step and developed a comprehensive mathematical framework for such a mass based model. Additionally in many clinical/laboratory test suites, only one concentration/volume location was used for measurements while further information was derived from general assumptions about humidity. Moreover, bedside tests tend to neglect the influence of the dead space in tubing and connectors on humidity measurements^13,14,17^. The dead space contributes always to moisture benefit and reduces water output. This effect is more pronounced with low HME performance.

### Volume effects in measurements

Adding water vapour to the inspired gas increases the gas volume. This obvious fact has been neglected [3, p 185] or ignored by other investigators up to now.

Our results show that the target humidity of 44 mg/l leads to a volume increase of about 6.6% with regard to the inspired dry gas. It can be seen without formal statistical treatment from Fig. 4 that the distribution of this additional volume between *V*_*mb*_ and *V*_*exp*_ depends on the HME performance. The higher the HME performance the more water vapour is present in the *V*_*mb*_.

Whether this difference of 6.6% is relevant in a clinical assessment remains open. In the laboratory test situation it is however mandatory to indicate the location of the volume measurement.

### HME evaluation procedures

We have applied the validated test rig to assess a selection of HMEs by measuring both, moisture benefit and water output together with the corresponding volumes. In Fig. 5a-b these measurements are depicted together with the corresponding base-line data for reference. The complete set of data comprising five HME types with three specimen each at three carrier gas volumes and two humidity levels shows the excellent reproducibility of the test arrangement. The measurements were considered to provide a solid basis for calculating the HME performance characteristics of the devices using the evaluation mode of the model (see appendix Fig. 11c). In Fig. 6 data pairs of 132 separate measurements are presented. Horizontal and vertical bars denote 5% mean uncertainty, since both ways of calculation propagate measurement errors in humidity and volume data. The uncertainty reflected in the scatter of the data points illustrates the limits of the plug flow model underlying the calculations.

### Flow profile dynamics

**Fig. 10:**
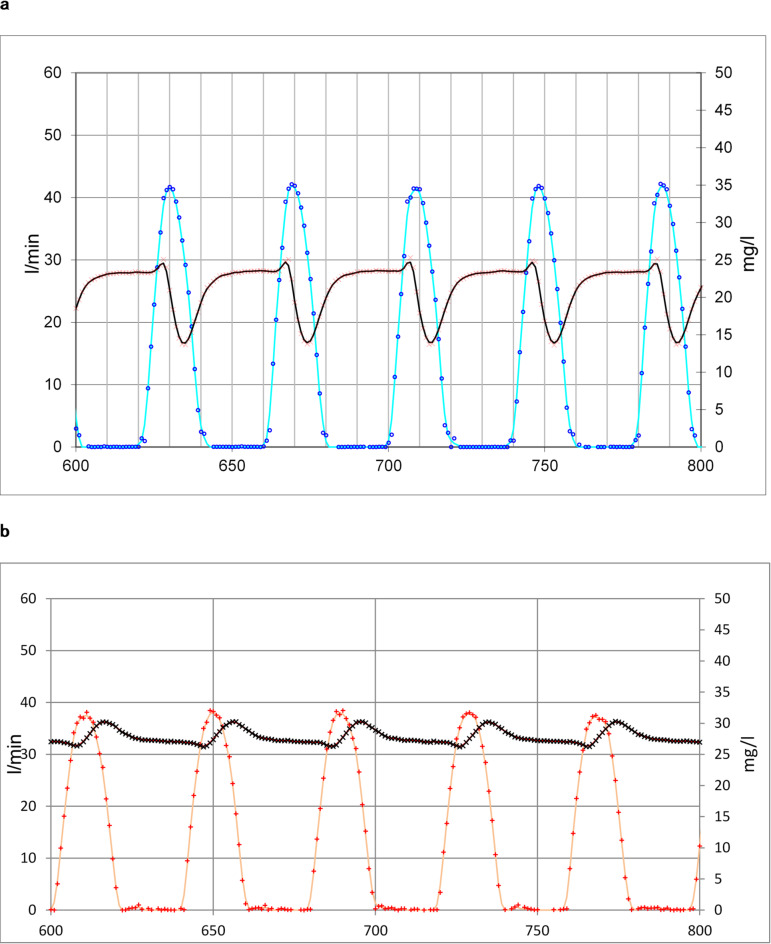
(**a**) Flow and humidity dynamics in steady state at water output location. Flow water output (L/min): dark blue points measurements, blue line fitted, Humidity water output (mg/L): red points measurements, black line fitted. (**b**) Flow and humidity dynamics in steady state at moisture benefit location. Flow moisture benefit (L/min): red points measurements, red line fitted, Humidity moisture benefit (mg/L): black points measurements, black line fitted.

The sinusoidal flow profile (see methods) is equivalent to the square wave profile provided the same gas volume is delivered. The humidity in the system oscillates considerably during a breathing cycle. This is more pronounced at the water output location compared to the moisture benefit branch. The humidity decreases for instance from 23.3 to 13.7 mg/l and returns back within 4 s in the water output branch (750 ml *V*_*exp*_, 44 mg/l target humidity). In contrast, under identical conditions the humidity amplitude for moisture benefit is only 3 mg/l. Turbulent flow and mixture effects are present in such a high dynamical situation. Such effects are not incorporated in a plug flow model. For the validity of the plug flow model approach, it is decisive that the steady state assumption is fulfilled (see Figs. 10a and b).

### HME performance and efficiency

Regardless to the choice of the location, measurement of one humidity and one volume parameter is sufficient to evaluate the full model. Our preference for moisture benefit and the corresponding volume is founded on the similarity to active humidification (HH) and the possibility to assess the performance of LE-HMEs with such a setup. Therefore, the results presented in Fig. 7 are based on measurements of moisture benefit and the corresponding volume *V*_*mb*_. Both quantities, HME performance and water load entering the HME during expiration cannot be calculated separately because the model in Fig. 1 constitutes a self-consistent dynamic system.

In the situation without HME a prediction of the model variables based on target humidity, tidal volume, dead space and *hb* can be obtained. With HME in-place information about the performance or the efficiency is additionally needed for predictions. The water load ($\:{m}_{e}^{ent}$) can be approximated with sufficient precision as the product of target humidity and volume *V*_*mb*_. Using the algorithms of the model framework for the evaluation of measurements all variables are calculated in one pass including humidification efficiency as the quotient of HME performance and the amount $\:{m}_{e}^{ent}$ - $\:{m}_{i}^{ent}$.

For some HMEs (#4, #5) in Fig. 7 the performance at first glance appears to increase linearly within the observation interval. These specimen possess in our opinion a sufficiently large total water retention capacity. In this case an overall efficiency may be obtained as the slope of a linear regression function. Other HMEs (#2, #3) show an asymptotic behaviour. Therefore, an overall efficiency does not exist. This metric must in contrast be estimated individually for each measurement scenario. A third type of HME (#1) shows an almost constant HME performance within the observation interval. We assume that here the maximum retention capacity is approached if not exceeded by the water load.

The discrimination between these three different HME types is further improved by using the combination of efficiency and *V*_*mb*_ (see Fig. 8). Apparently, HME performance and water load vary not entirely co-linear. The subtle effects of dead space and surface influences of the housing in addition to adsorption-desorption dynamics may explain this. In any case, the HME efficiency gives the fraction of the amount of water presented which is returned from the HME. The diagram in Fig. 8 can be regarded as a useful tool to select an HME product for particular clinical applications. A combination of efficiency threshold and tidal volume limit can be employed to comprehensively describe the humidification properties of a device. The efficiency registered at the volume limit is therefore guaranteed over the whole observation range. For practical purposes the efficiency at the upper bound specified by the manufacturer is needed.

It is understood that in addition to HME efficiency other criteria namely dead space, resistance, filtration properties and last but not least, cost will play a vital role in HME selection. This is beyond the scope of this work.

### LE-HMEs

While a multitude of publications is concerned with conventional HMEs in a variety of laboratory and clinical experimental situations only few papers discuss the evaluation of water retention capacity^2^ and efficiency in LE-HMEs^16^. Grolman and coworkers have applied a modified version of the ISO test method published in 1992 to LE-HMEs and report moisture output results between 20.1 and 24.7 mg/l. The term moisture output as used in this ISO 9360 edition is approximately equivalent to moisture benefit (*mb*) in Fig. 2. We have used the data published by Grolman et al. to calculate (see appendix) the corresponding HME performance as 5.5 to 7.5 mg per breath or 42–50% efficiency. In contrast, van de Boer and associates developed an ex-vivo method for the measurement of HME performance to overcome the complexities found in the application of the ISO measurement procedure. Their findings amount to HME performances between 1 and 5 mg in the average breathing range from 0.1 to 0.5 l tidal volume. Comparison of these previously published data with our measurements in Fig. 9 reveals the difficulties present in the field of HME evaluation endeavour. Terms and methods cannot easily be entirely matched. Nevertheless, our results appear to corroborate the numbers in the work of van den Boer while the calculated performance results based on the ISO standard method adapted by Grolman seem to positively biased. Especially in the case of LE-HMEs quality of life aspects are of paramount importance which in turn are affected by criteria like ease of use, duration of use, cost and more. Again, our focus is only on the water retention properties of HMEs.

## Conclusion

Based on the results presented in this work we claim that an HME tested with a qualified test system in the way described will under defined conditions show consistent efficiency in steady state. The device will then store and return an amount of water that is a fraction of the amount delivered. This fraction will mainly depend on tidal volume and is also influenced by the target humidity. We suggest that the reported tidal volume should always refer to the inspired volume with defined humidity, which is readily available or can easily be calculated. A comprehensive self-consistent set of functions in a mathematical framework has been developed for all required calculations, both evaluations and predictions including scenarios with external humidity (*hb*) greater than 0. The combination of efficiency and corresponding tidal volume can now be set as a basis for informed choice of HME. This novel approach could even serve as a solid base for a future revision of the international standard.

## Supplementary Information

Below is the link to the electronic supplementary material.

Supplementary Material 1

## Data Availability

The data presented in the current study are available from the corresponding author on reasonable request.

## Abbreviations

eta: $$\:\eta\:$$, efficiency
ha: Target humidity
hb: Humidity carrier gas
HME: Heat and moisture exchanger
LE-HME: Heat and moisture exchanger for laryngectomized patients
mb: Moisture benefit
$\:{m}_{e}^{ent}$: Mass entering during expiration
$\:{m}_{e}^{ext}$: Mass exiting during expiration
$\:{m}_{i}^{ext}$: Mass exiting during inspiration
$\:{m}_{i}^{ent}$: Mass entering during inspiration
$$\:{m}_{}^{ret}$$: Mass returned
$$\:{m}_{}^{sup}$$: Mass supplied
$$\:{m}_{}^{st}$$: Mass stored
V_D_: Dead space
V_exp_: Volume expired
V_insp_: Volume inspired
V_mb_: Volume returned
wo: Water output
