# Supplementary material for: Comprehensive structural model for the evaluation of HME humidification properties
Source: Sci Rep. 2025 Sep 17;15:32569. doi: 10.1038/s41598-025-17916-z (PMC12443951; doi:10.1038/s41598-025-17916-z)
Supplement: Supplementary file 1 — Supplementary Material 1 [file 41598_2025_17916_MOESM1_ESM.docx]

**Appendix**

**HME calculator**

The complete set of routines was incorporated into a simple browser based computer program to facilitate predictions and evaluations of measurements. This useful tool can in addition be employed to perform what-if-simulations.


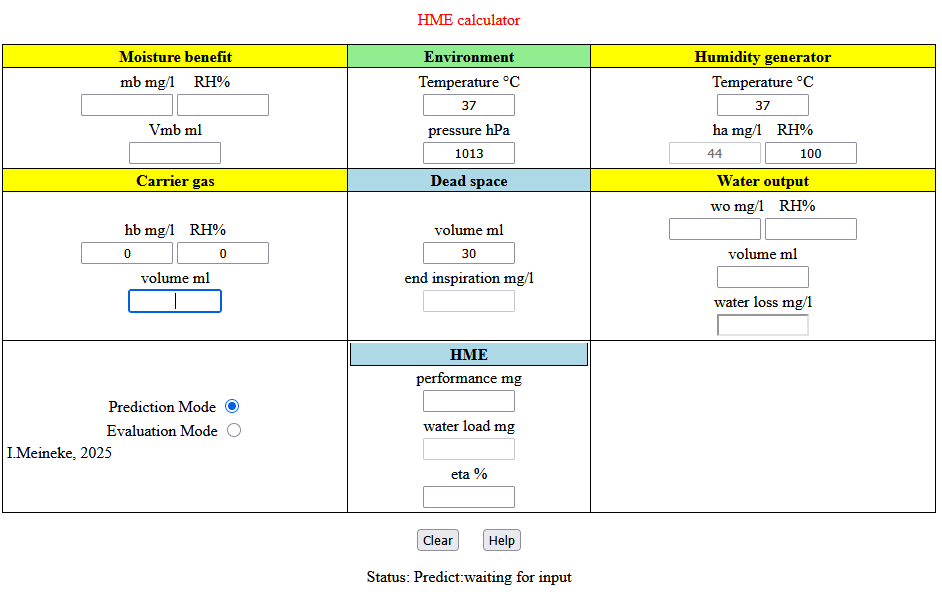


Fig. 11a HME calculator screen in prediction mode without HME awaiting input

**
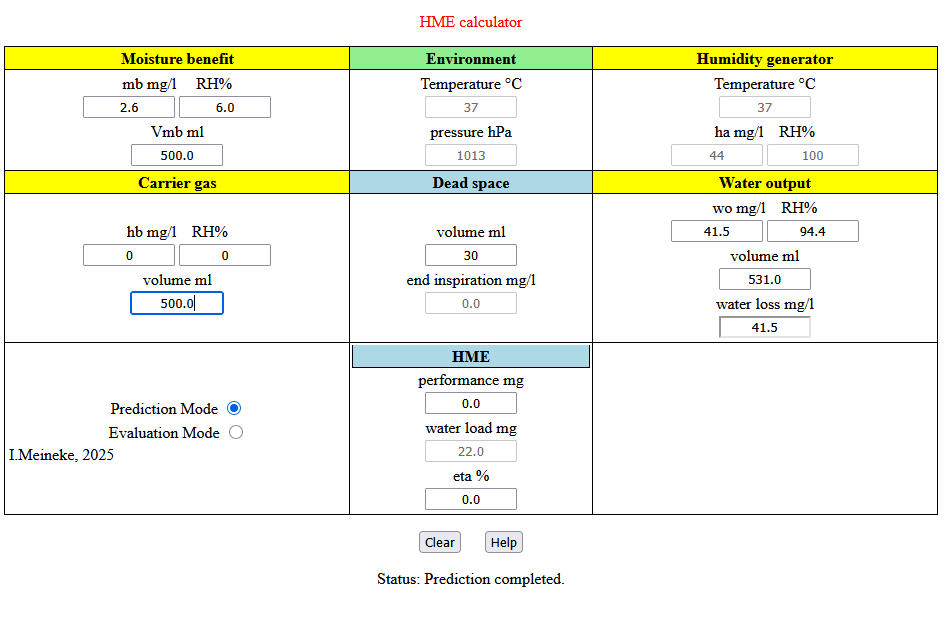
**

Fig. 11b HME calculator screen in prediction mode without HME after input *V_insp_* 500ml.

**
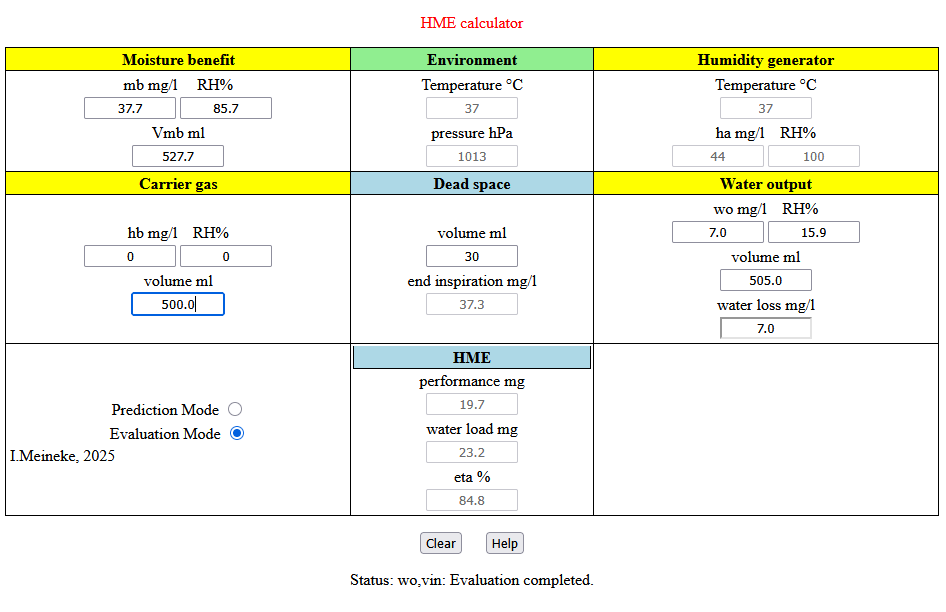
**

Fig. 11c HME calculator screen in evaluation mode with HME after input *V_insp_* 500ml with water output 7mg/l. The program is available from the authors upon request.

**Prediction and evaluation equations**

In the following derivations, masses and volumes are understood as molar and concentrations as molar fractions at given temperature and pressure respectively. The calculations rely on the central humidification assertion which simply states that the gas exiting the humidifier (see fig. 1) is water vapour saturated at the preselected temperature in terms of absolute humidity.

1 $h_{a}=\frac{m^{ret}+m_{e}^{ext}-m_{i}^{ent}}{V_{mb}+m_{e}^{ext}-m_{i}^{ent}}$

The remaining basic equations are stated below with the additional definitions of $m_{i}^{prev}$ (amount remaining in the dead space at the end of expiration) and $m_{e}^{prev}$ (amount remaining in the dead space at the end of inspiration):

2 $m^{ret}=mb*V_{mb}$

3 $m_{e}^{ext}=wo*V_{exp}$

4 $m_{i}^{ent}= hb*V_{insp}$

5 $m_{i}^{prev}=ha*V_{D}$

6 $m_{i}^{ext}=m_{i}^{ent}+m^{st}$

7 $m_{e}^{prev}=m_{i}^{ext}*\frac{V_{D}}{V_{mb}}$

8 $m^{ret}=m_{i}^{ext}+ m_{i}^{prev}-m_{e}^{prev}$

9 $m^{sup}=m^{ret}+m_{e}^{ext}-m_{i}^{ent}$

10 $m_{e}^{ent}=m^{sup}+m_{e}^{prev}-m_{i}^{prev}$

11 $V_{mb}=V_{insp}+m^{st}$

12 $V_{exp}=V_{insp}+m_{e}^{exp}-m_{i}^{ent}=V_{insp}+wo*V_{exp}-h_{b}*V_{insp}$

13 $m^{add}=m_{e}^{ext}-m_{i}^{ent}$

14 $m^{net}=m_{e}^{ent}-m_{i}^{ent}$

Subsequent calculations depend on the task at hand, i.e. prediction of test rig data or evaluation of HME measurements, and the humidity and volume information available.

**Predictions**

Predictions assume that the HME performance is known.

A. Prediction of test rig status from volume *V_insp_*_:_

Input: $ha$, $hb$, $V_{D}, V_{insp}$, $m^{st}$

Several terms can directly calculated from input:

$$m_{i}^{ext}= hb*V_{insp}$$

$$m_{i}^{prev}=ha*V_{D}$$

$$V_{mb}=V_{insp}+m^{st}$$

$$m_{i}^{ext}=m_{i}^{ent}+m^{st}$$

$$m_{e}^{prev}=m_{i}^{ext}*\frac{V_{D}}{V_{mb}}$$

$$m^{ret}=m_{i}^{ext}+m_{i}^{prev}-m_{e}^{prev}$$

The humidification assertion is rearranged to obtain $m_{e}^{ext}$:

$$ha=\frac{m^{ret}+m_{e}^{ext}-m_{i}^{ent}}{V_{mb}+m_{e}^{ext}-m_{i}^{ent}}$$

$$ha*V_{mb}+ha*m_{e}^{ext}-ha*m_{i}^{ent}=m^{ret}+m_{e}^{ext}-m_{i}^{ent}$$

$$ha*V_{mb}-ha*m_{i}^{ent}+m_{i}^{ent}=m^{ret}-ha*m_{e}^{ext}+m_{e}^{ext}$$

$$ha*V_{mb}+m_{i}^{ent}*\left( 1-ha \right)=m^{ret}+m_{e}^{ext}*(1-ha)$$

$$m_{e}^{ext}=\frac{ha*V_{mb}-m^{ret}}{1-ha}+m_{i}^{ent}$$

The remaining variables can be calculated.

$$m^{sup}=m^{ret}+m_{e}^{ext}-m_{i}^{ent}$$

$$m_{e}^{ent}=m^{sup}-m_{i}^{prev}+m_{e}^{prev}$$

$$V_{exp}=V_{insp}+m_{e}^{ext}-m_{i}^{ent}$$

$$eta=\eta=\frac{m^{st}}{(m_{e}^{ent}-m_{i}^{ent})}$$

$$mb=m^{ret}/V_{mb}$$

$$wo=m_{e}^{ext}/V_{exp}$$

All variables can then be converted into SI units.

B. Prediction of test rig status from volume *V_mb_*

Input: $ha$, $hb$, $V_{D}, V_{mb}$, $m^{st}$

$$V_{insp}=V_{mb}-m^{st}$$

The calculation follows the steps outlined under A.

C. Prediction of test rig status from volume *V_exp_*_:_

Input: $ha$, $hb$, $V_{D}, V_{exp}$, $m^{st}$

Starting with the humidification assertion and using basic definitions

$$ha=\frac{m^{ret}+m_{e}^{ext}-m_{i}^{ent}}{V_{exp}+m^{st}}=\frac{m^{st}+m_{i}^{prev}-m_{e}^{prev}+m_{e}^{ext}}{V_{exp}+m^{st}}$$

$m_{e}^{ext}=m_{i}^{ent}+m^{add}$ , $m_{i}^{ent}=hb*V_{insp}=hb*(V_{exp}-m^{add})$

the humidification assertion is rearranged.

$$ha*\left( V_{exp}+m^{st}-V_{D} \right)-m^{st}=m_{e}^{ext}-m_{e}^{prev}=V_{exp}*hb+m^{add}*\left( 1-hb \right)-m_{e}^{prev}$$

This results in an expression for $m_{e}^{prev}$.

$$m_{e}^{prev}=V_{exp}*hb+m^{add}*\left( 1-hb \right)+m^{st}-ha*\left( V_{exp}+m^{st}-V_{D} \right)$$

With a second expression for $m_{e}^{prev}$ the system can be solved.

$$m_{e}^{prev}=m_{i}^{ext}*\frac{V_{D}}{V_{mb}}=\frac{\left[ \left( V_{exp}-m^{add} \right)*hb+m^{st} \right]*V_{D}}{V_{exp}-m^{add}+m^{st}}$$

Solve for $m^{add}$ letting:

$$a=m^{st}+hb*V_{exp}-ha*(V_{exp}+m^{st}-V_{D})$$

$$b=(hb*V_{exp}+m^{st})*V_{D}$$

$$c=hb*V_{D}$$

$$d=V_{exp}+m^{st}$$

$${m^{add}}^{2}-\left( \frac{a+c}{1-hb}+d \right)*m^{add}+\frac{a*d+b}{1-hb}=0$$

The remaining variables can be calculated subsequently.

$$V_{insp}=V_{exp}-m^{add}$$

$$m_{i}^{ent}=hb*V_{insp}$$

$$V_{mb}=V_{insp}+m^{st}$$

$$m_{i}^{ext}=m_{i}^{ent}+m^{st}$$

$$m_{i}^{prev}=ha*V_{D}$$

$$m_{e}^{prev}=m_{i}^{ext}*\frac{V_{D}}{V_{mb}}$$

$$m^{ret}=m_{i}^{ext}+m_{i}^{prev}-m_{e}^{prev}$$

$$m^{sup}=m^{ret}+m_{e}^{ext}-m_{i}^{ent}$$

$$m_{e}^{ent}=m^{sup}-m_{i}^{prev}+m_{e}^{prev}$$

$$m_{e}^{ext}=m_{e}^{ent}-m^{st}$$

$$eta=\eta=\frac{m^{st}}{(m_{e}^{ent}-m_{i}^{ent})}$$

$$mb=m^{ret}/V_{mb}$$

$$wo=m_{e}^{ext}/V_{exp}$$

All variables can then be converted into SI units.

**Evaluations**

Evaluations require that one concentration and one volume measurement are available.

A. Evaluation of HME performance and efficiency from concentration *mb* and volume *V_mb_*

Input: $ha$, $hb$, $V_{D}$, *mb*$, V_{mb}$

Using the basic definitions

$$m^{ret}=mb*V_{mb}$$

$$m_{i}^{ext}=m^{ret}-m_{i}^{prev}+m_{e}^{prev}$$

$$m_{e}^{prev}=m_{i}^{ex}*\frac{V_{D}}{V_{mb}}$$

and substituting for $m_{e}^{prev}$

$$m_{i}^{ext}=(m^{ret}-m_{i}^{prev})/(1-\frac{V_{D}}{V_{mb}})$$

$$m_{i}^{ent}=hb*V_{insp}=hb*(V_{mb}-m^{st})$$

the HME performance is readily available.

$$m^{st}=m_{i}^{ext}-m_{i}^{ent}=\frac{m^{ret}-m_{i}^{prev}}{1-\frac{V_{D}}{V_{mb}}}-hb*V_{mb}+hb*m^{st}$$

$$m^{st}*(1-hb)=\frac{m^{ret}-m_{i}^{prev}}{1-\frac{V_{D}}{V_{mb}}}-hb*V_{mb}$$

$m^{st}$ and the remaining variables can subsequently be calculated.

$$m_{i}^{ext}=m_{i}^{ent}+m^{st}$$

$$m_{i}^{prev}=ha*V_{D}$$

$$m_{e}^{prev}=m_{i}^{ext}*\frac{V_{D}}{V_{mb}}$$

$$m^{ret}=m_{i}^{ext}+m_{i}^{prev}-m_{e}^{prev}$$

$$m^{sup}=m^{ret}+m_{e}^{ext}-m_{i}^{ent}$$

$$m_{e}^{ent}=m^{sup}-m_{i}^{prev}+m_{e}^{prev}$$

$$m_{e}^{ext}=m_{e}^{ent}-m^{st}$$

$$eta=\eta=\frac{m^{st}}{(m_{e}^{ent}-m_{i}^{ent})}$$

$$mb=m^{ret}/V_{mb}$$

$$wo=m_{e}^{ext}/V_{exp}$$

All variables can then be converted into SI units.

B. Evaluation of HME performance and efficiency from concentration *wo* and volume *V_exp_*

Input: $ha$, $hb$, $V_{D},$ $V_{exp},$ $wo$

Starting with the humidification assertion:

$$ha=\frac{m^{st}+m_{i}^{prev}-m_{e}^{prev}+m_{e}^{ex}}{V_{exp}+m^{st}}$$

with $m_{e}^{prev}=m_{i}^{ex}*\frac{V_{D}}{V_{mb}}$ expanded to $m_{e}^{prev}=\left( m_{i}^{en}+m^{st} \right)*V_{D}/(V_{insp}+m^{st})$

rearrangement gives

$$ha*\left( V_{exp}+m^{st} \right)=m^{st}+m_{i}^{prev}+m_{e}^{ex}-\left( m_{i}^{en}+m^{st} \right)*V_{D}/(V_{insp}+m^{st)}$$

using $V_{insp}=V_{exp}*(1-wo)/(1-hb)$ and letting

$$b=(V_{insp}*\left( 1-ha \right)-V_{exp}*ha-V_{D}+m_{e}^{exp}+m_{i}^{prev})/(1-ha)$$

$$c=(\left( m_{e}^{ex}+m_{i}^{prev} \right)*V_{insp}-m_{i}^{en}*V_{D}-V_{insp}*V_{exp}*ha)/(1-ha)$$

the system can be solved for $m^{st}$.

$$m^{{st}^{2}}+b*m^{st}+c=0$$

The remaining variables can be obtained subsequently.

$$V_{mb}=V_{insp}+m^{st}$$

$$m_{i}^{ent}=hb*V_{insp}$$

$$m_{i}^{ext}=m_{i}^{ent}+m^{st}$$

$$m_{e}^{prev}=m_{i}^{ext}*\frac{V_{D}}{V_{mb}}$$

$$m^{ret}=m_{i}^{ext}+m_{i}^{prev}-m_{e}^{prev}$$

$$m^{sup}=m^{ret}+m_{e}^{ext}-m_{i}^{ent}$$

$$m_{e}^{ent}=m^{sup}-m_{i}^{prev}+m_{e}^{prev}$$

$$m_{e}^{ext}=m_{e}^{ent}-m^{st}$$

$$eta=\eta=\frac{m^{st}}{(m_{e}^{ent}-m_{i}^{ent})}$$

$$mb=m^{ret}/V_{mb}$$

$$wo=m_{e}^{ext}/V_{exp}$$

All variables can then be converted into SI units.
